# Distributed Optical Fiber Sensors Based on Optical Frequency Domain Reflectometry: A review

**DOI:** 10.3390/s18041072

**Published:** 2018-04-03

**Authors:** Zhenyang Ding, Chenhuan Wang, Kun Liu, Junfeng Jiang, Di Yang, Guanyi Pan, Zelin Pu, Tiegen Liu

**Affiliations:** 1 School of Precision Instrument & Optoelectronics Engineering, Tianjin University, Tianjin 300072, China; Wang_chen_huan@163.com (C.W.); jiangjfjxu@tju.edu.cn (J.J.); style80230808@163.com (D.Y.); guanyipan@126.com (G.P.); 15222607356@163.com (Z.P.); tgliu@tju.edu.cn (T.L.); 2 Key Laboratory of Optoelectronics Information Technology, Ministry of Education, Tianjin 300072, China; 3 Tianjin Optical Fiber Sensing Engineering Center, Institute of Optical Fiber Sensing of Tianjin University, Tianjin 300072, China

**Keywords:** optical frequency domain reflectometry (OFDR), distributed optical fiber sensors, Rayleigh scattering, optical fiber sensors

## Abstract

Distributed optical fiber sensors (DOFS) offer unprecedented features, the most unique one of which is the ability of monitoring variations of the physical and chemical parameters with spatial continuity along the fiber. Among all these distributed sensing techniques, optical frequency domain reflectometry (OFDR) has been given tremendous attention because of its high spatial resolution and large dynamic range. In addition, DOFS based on OFDR have been used to sense many parameters. In this review, we will survey the key technologies for improving sensing range, spatial resolution and sensing performance in DOFS based on OFDR. We also introduce the sensing mechanisms and the applications of DOFS based on OFDR including strain, stress, vibration, temperature, 3D shape, flow, refractive index, magnetic field, radiation, gas and so on.

## 1. Introduction

Distributed optical fiber sensors (DOFS) can measure lots of sensing parameters distributedly along the fiber under test (FUT) such as strain, stress, vibration, temperature, 3D shape, flow rate, refractive index, magnetic field, radiation, gas, etc. DOFS have many attracting features such as light weight, small size, non-corrosive, immunity to electromagnetic interference, harsh environment suitability, large scale, and high sensing density, which are very useful for many sensing applications [1,2]. 

Several DOFS technologies have been developed based on the measurement of intrinsic backscattering, including Raman, Brillouin and Rayleigh scattering [1], which are based on optical time domain reflectometry (OTDR) and optical frequency domain reflectometry (OFDR). The Rayleigh scattering-based DOFS is usually classified into two categories: OTDR and OFDR. OTDR is a common technology with wide application in the optical fiber communication and sensing field. In OTDR, the input pulse generated is launched into the fiber under test (FUT). As the input pulse propagates along the FUT, the light from the pulse that gets back-reflected light either via Rayleigh backscattering or Fresnel reflection is then measured by using a photo-detector. The sensing range to the point of reflection is dependent on the time delay between the input light pulse and the corresponding back-reflected light. However, the key parameters of the signal-to-noise-ratio (SNR), the sensing range and the sensing spatial resolution in OTDR need to be balanced [3,4,5,6,7]. For example, if the spatial resolution need to be improved, the width of the input pulse need to be decreased and the bandwidth of the photo-detectors need to be increased. The sensing range will be deteriorated due to the energy weaken by a short pulse width of the input light. The SNR will be also decreased by increasing bandwidth of the photo-detectors. In addition, the peak pulse power of input light in OTDR cannot be increased greatly to restrain the nonlinear effects in FUT [8].

Among all the distributed sensing techniques, OFDR has been given tremendous attention because of its high spatial resolution and large dynamic range. Eickhoff et al. firstly presented the OFDR method by using Rayleigh backscattering of an optical fiber in 1981, which is similar to the technology of frequency modulated continuous wave (FMCW) radar [9]. A basic OFDR configuration consists of a tunable laser source (TLS) which optical frequencies can be tuned linearly in time without any mode hops and an interferometer that comprises a test path and a reference path. The reference path is considered as a local (LO) oscillator whereas the FUT is connected to the test path. Interferences are generated between the LO signals and back-reflected light in FUT coming from the test path that contains Rayleigh backscattering and Fresnel reflection. The beat frequencies are obtained by a Fourier transform applying to the interferences signals. If the tuning rate of the TLS is a constant, the beat frequencies are proportional to the length of FUT [10]. 

OFDR is firstly applied to test the components and assemblies in the optical fiber networks at a short range as an order of tens to hundreds of meters, but the spatial resolution of OFDR can range from up to ten micrometers to several millimeters [11,12,13,14]. Along with the technological progress of the narrow linewidth of TLS and compensation methods of nonlinear phase noise in OFDR, the sensing range can be up to an order of hundred kilometers [15,16,17,18]. In the distributed sensing methods based on OFDR, in 1998, Froggatt et al. present a distributed high sensitivity strain and temperature sensing method with a spatial resolution at an order of millimeters to centimeters using Rayleigh backscattering spectra (RBS) shifts in OFDR [19,20]. Rayleigh scattering is generated by random refractive index variations along a FUT, and it can be considered as a distributed, weak fiber Bragg grating (FBG) with random periods. The strain or temperature variations on FUT can result in a local RBS spectral shifts and they are obtained from the cross-correlation between two RBS measurements. One is as a measured RBS and the other is as a referential RBS. Now the sensing parameters in OFDR-based distributed sensing methods have been extended to strain, stress, vibration, temperature, 3D shape, flow, refractive index, magnetic field, gas and so on.

In this review, we survey the compensation of nonlinear phase noise in OFDR as a key technology for improving the sensing range, the spatial resolution and sensing performances in DOFS based on OFDR. We also introduce the sensing mechanisms and the applications of DOFS based on OFDR including strain, stress, vibration, temperature, 3D shape, flow, refractive index, magnetic field, radiation and gas.

## 2. The Principle of OFDR 

**Figure 1:**
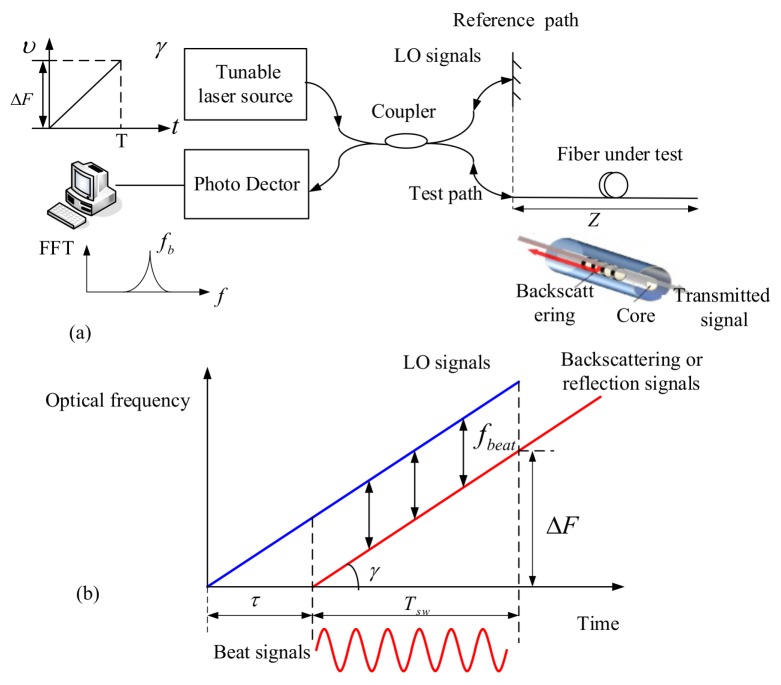
(**a**) OFDR basic configuration and (**b**) linearly tuning optical frequency and beating signals come from the LO light and received light.

The beat interference between two light signals comes from the same linear tuning TLS. The basic configuration is shown in Figure 1a. One signal described by an electric field intensity $E_{s}(t)$ is back-reflected light in the FUT that contains Rayleigh backscattering and Fresnel reflection along the test path, whereas, another signal described by an electric field intensity $E_{r}(t)$ comes from the reference path. Assuming that the tuning rate of the TLS is γ, the optical field $E_{r}(t)$ coming from the reference path can be given by [21]:

(1) $$E_{r}(t)=E_{0}\exp\left\{j[2\pi f_{0}t+\pi \gamma t^{2}+e(t)]\right\},$$

where $f_{0}$ is an initial optical frequency. e(t) is phase noise or nonlinear phase. $E_{s}(t)$ can be given by:

(2) $$E_{s}(t)=\sqrt{R(\tau )}E_{0}\exp\left\{j[2\pi f_{0}(t-\tau )+\pi \gamma {\left(t-\tau \right)}^{2}+e(t-\tau )\right\},$$

where R(τ) is reflectivity of FUT at the time delay τ. $E_{r}(t)$ and $E_{s}(t)$ are the LO (reference path) and received light signals (test path), respectively. The beating signals with intensity I(t) coming from the interference of $E_{r}(t)$ and $E_{s}(t)$ are shown in Figure 1b. I(t) is written as:

(3) $$I(t)=2\sqrt{R(\tau )}E_{0}^{2}\cos\left\{2\pi [f_{0}\tau +\gamma \tau t+\frac{1}{2}\gamma \tau^{2}+e(t)-e(t-\tau )]\right\},$$

where the beat frequency $f_{b}=\gamma \tau$ and $f_{b}$ are in proportionality to the length of FUT. The last term e(t)−e(t−τ) is the phase noise or nonlinear phase in the beating signals.

## 3. Compensation of Nonlinear Phase Noise in OFDR 

### 3.1. Nonlinear Phase Noise in OFDR

**Figure 2:**
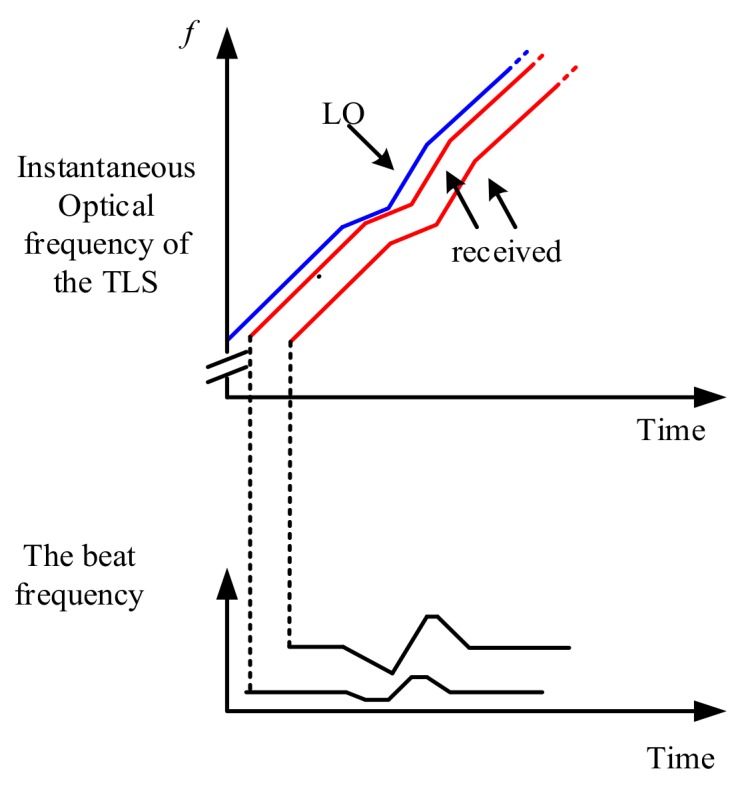
Beating signals are generated from the LO light and received light under the condition of the nonlinear optical frequency tuning of a TLS.

In OFDR, the beating signals are collected in the optical frequency domain. A Fast Fourier transform (FFT) is then utilized to convert the optical frequency domain signals to the spatial domain signals. A nonlinear phase in the beating signals results in a spreading of the back-reflected light energy, decreasing of the refection intensity and worsening of the spatial resolution [10,22,23,24]. Figure 2 shows that beating signals interference between the received light and LO light, which are sampled under the influence of a nonlinear tuning of TLS. The beat frequencies are not constants and many other frequencies are generated. Namely, the reflection peaks will be widened in the spatial domain. Above all, the compensation of nonlinear phase in OFDR is a key technology for improving the sensing range, the spatial resolution and the sensing performances of OFDR.

### 3.2. Frequency-Sampling Method for Compensation of Nonlinear Phase Noise in OFDR

**Figure 3:**
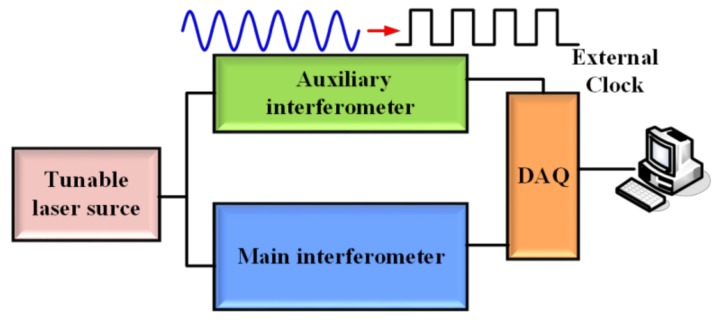
Frequency-sampling method for compensation of nonlinear phase noise in OFDR, DAQ is a data acquisition card.

Three types of the nonlinear phase compensation methods are applied to solve this issue. One is a frequency-sampling method. The second is software algorithms for compensation of nonlinear phase in OFDR. The third is a short tuning range of TLS based method. In the frequency-sampling method, the data acquisition card of OFDR samples the beating signals at even optical frequency intervals. DAQ’s external clock is triggered by “optical frequency” clock generated by an auxiliary interferometer shown in Figure 3 [10,22]. However, a shortcoming of this method is that the maximum sensing range is restricted by the length of delay fiber in the auxiliary interferometer due to Nyquist Law, so the sensing range of this frequency-sampling method is short [14]. In order not to increase the length of delay fiber in the auxiliary interferometer, the sensing range of OFDR can be increased by a frequency multiplication method. However, the sensing range enhancement is still influenced by the frequency multiplication components and the “optical frequency” clock jitter [25]. 

### 3.3. Software Algorithm for Compensation of Nonlinear Phase Noise in OFDR

**Figure 4:**
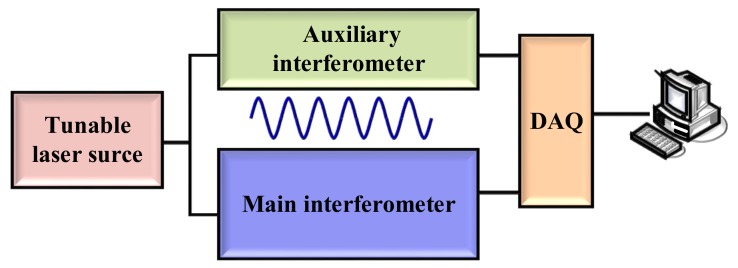
Software algorithms for compensation of the nonlinear phase noise in OFDR. DAQ is a data acquisition card.

The second type of the method is to utilize some algorithms to compensate the nonlinear phase in OFDR after the data acquisition. In this type method, an auxiliary interferometer is utilized to acquire the phase information of the TLS and several kinds of software algorithms are implemented to compensate the nonlinear phase shown in Figure 4. 

One kind of these software algorithms is the re-sampling method. In this method, the interpolation algorithms re-sample the main interference signals with uniform optical frequency intervals based on the TLS’s phase change. The interpolation algorithms include the linear, cubic spline interpolation [10,26,27,28] and non-uniform FFT (NUFFT) [29]. The merit of re-sampling method is that the maximum measurement distance is no longer restricted by the length of delay fiber in the auxiliary interferometer. 

#### 3.3.1. NUFFT

**Figure 5:**
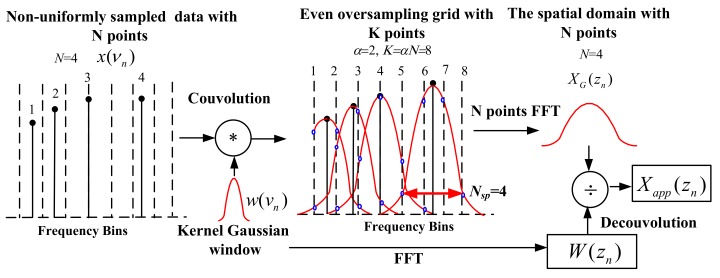
Signal processing procedure of NUFFT (Reprinted from Ding et al. [29]. with permission of AIP Publishing LLC; copyright (2012)).

**Figure 6:**
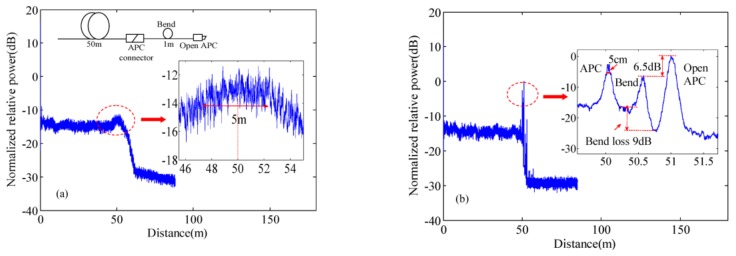
Comparison between OFDR traces for 51 m FUT using (**a**) standard FFT and (**b**) NUFFT (Reprinted from Ding et al. [29] with permission of AIP Publishing LLC; copyright (2012)).

NUFFT can be implemented to compensate the nonlinear phase in OFDR [29]. The NUFFT processing procedure is shown in Figure 5. In NUFFT, the original non-uniformly sampled signals *x*(*ν*_n_) in the optical frequency domain are shown by solid circles and the uniform grid with α = 2 oversampling ratio are shown by vertical dashed lines. *x*(*ν*_n_) is convolved with a Gaussian window function *w*(*ν*_n_) and *x*(*ν*_n_) spreads its energy over adjacent uniform grids with *N*_sp_ = 4 points, as shown by the hollow circles. The summation of the values of the hollow circles on each dashed line is *x*(*ν*_k_) with new uniform intervals in the optical frequency domain. Then *x*(*ν*_k_) can be transformed to the spatial domain *X*_G_(*z*_n_) by a standard *N* points FFT. *X*_G_(*z*_n_) is deconvoluted by *W*(*z*_n_) and obtains *X*_app_(*z*_n_). Figure 6 shows that the OFDR traces in the spatial domain of testing a 50 m long FUT connected to a 1 m single mode fiber (SMF) pigtail. The Fresnel reflections due to two APC (angled physical contact) connectors of an optical fiber pigtail. The fiber pigtail is bended as a circle with a diameter of 1 cm. The peaks caused by APC connectors and bend cannot be discriminated by a standard FFT due to the nonlinear phase in OFDR shown in Figure 6a. Whereas, these peaks can be discriminated by NUFFT with a spatial resolution of less than 5 cm in Figure 6b. 

#### 3.3.2. Cubic Spline Interpolation

Song et al. present a cubic spline interpolation for the nonlinear phase compensation to increase the sensing range in OFDR with a high sensing resolution and accuracy [27]. They re-sample the main interference signals with uniform optical frequency intervals based on the TLS’s phase change by a cubic spline interpolation algorithm. This method can realize a theoretical spatial resolution of 0.3 mm and a SMF sensing range of over 300 m. Their OFDR system can also realize a distributed sensing with a strain and temperature resolution of 2.3 με and 0.7 °C in a spatial resolution of 7 cm, respectively.

#### 3.3.3. Concatenately Generated Phase Method 

**Figure 7:**
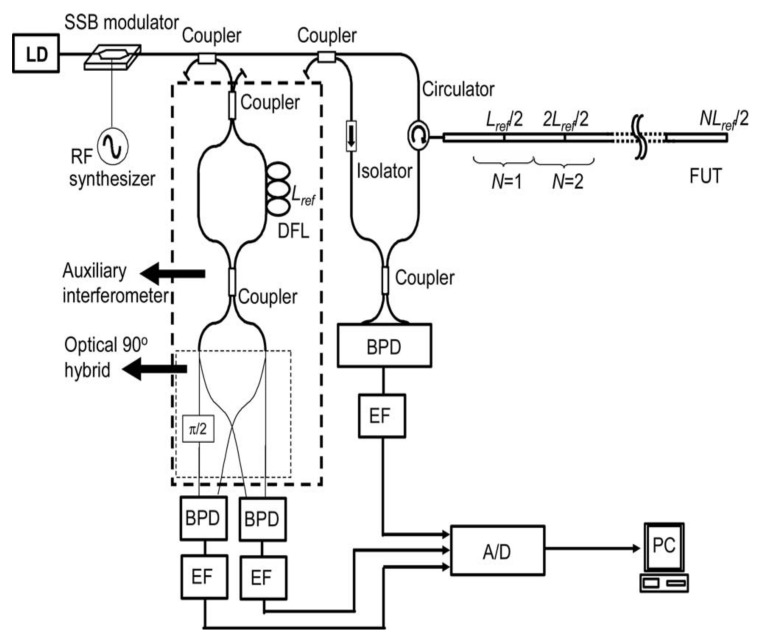
Experimental setup for CGPs nonlinear phase compensation method SSB is single sideband. DFL is delay fiber loop. *L_ref_* is the length of delay fiber in auxiliary interferometer. BPD is balanced photo-detector. EF is electrical filter. PC is personal computer (Reprinted from Fan et al. [32] with permission of OSA; copyright (2007)).

Fumihiko et al. presented a concatenately generated phase method to compensate the nonlinear phase noise and realize a 40 km OFDR system with a spatial resolution of 5 cm [30,31,32]. One of the key technologies in this method is a TLS with a high linear tuning using an external single sideband modulator [33] shown in Figure 7. The other of the key technologies of this method is to utilize a novel software algorithm for compensation of the nonlinear phase in OFDR, namely a concatenately generated phase method. The main interference signals are compensated by the reference signals acquired from an auxiliary interferometer. The reference signals from an auxiliary interferometer with *N*_ref_ delay time (*N* is an integer) in one arm can be expressed as:

(4) $$I_{N\mathrm{ref}}\propto \cos[2\pi \gamma N\tau_{ref}t+\theta (t)-\theta (t-N\tau_{ref})+C_{N\mathrm{ref}}]=\cos[X_{N}(t)].$$

where *C_Nref_* is a constant. *X_N_ (t)* is the phase term of *I_Nref_* and meet the following relationship:

(5) $$X_{N}(t)=\sum_{n=0}^{N-1}X_{1}\left(t-n\tau_{ref}\right),$$

which is called a “concatenately generated phase” (CGP) [32]. While the main interference beating signals are sampled based on timing corresponding to its constant increments, the phase noise is compensated as *τ_ref_* approaches *Nτ_ref_*. The FUT is separated into several segments and use different CGPs to compensate the nonlinear phase of the different segments in OFDR.

Using this method, they can measure a 40 km FUT. The spatial resolution of the peak at 40 km is improved 20 times than without any nonlinear phase compensation. Although CGPs method improves the measurement distance and the spatial resolution greatly, they are complicated and the process time is about 10 min [30].

#### 3.3.4. Deskew Filter

Ding et al. compensated the nonlinear phase in OFDR using an optimized deskew filter method [21,24,34]. The deskew filter is firstly utilized to solve the issue of the nonlinear frequency chirp in a FMCW Synthetic Aperture Radar (SAR) [35,36]. In the nonlinear phase compensation of OFDR, TLS’s phase change can be acquired from an auxiliary interferometer. The deskew filter method is utilized to compensate the nonlinear phase of the beating signals in the main interferometer by one time processing. In theory, this method could completely compensate any nonlinear phase provided that it can be acquired accurately, which can be restored by the signals collected form the auxiliary interferometer.

**Figure 8:**
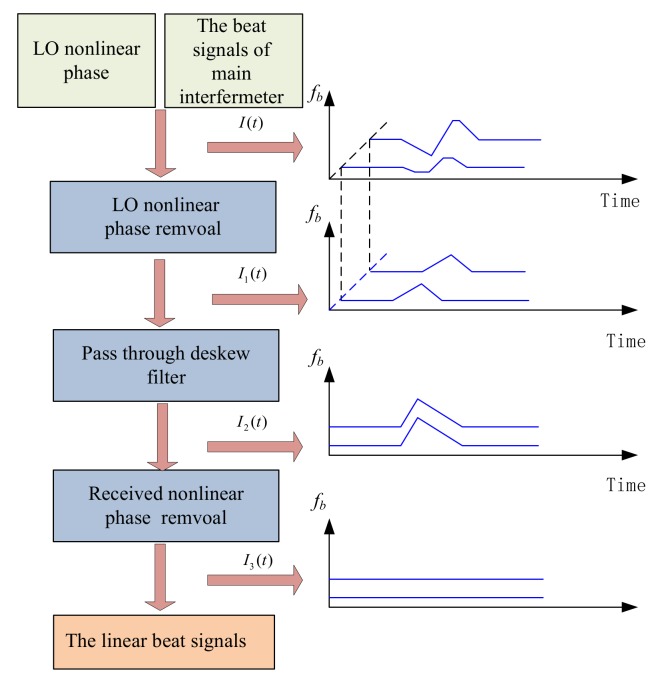
Signal processing procedure of the nonlinear phase compensation algorithm based on optimized deskew filter method (reprinted from Ding et al. [34] with permission of SPIE; copyright (2014)).

The theory of the nonlinear phase noise compensation using a deskew filter has already been described in detail [21,24,34]. We just provide a brief introduction here. The signal processing procedures of a nonlinear phase compensation algorithm based on the optimized deskew filter method are shown in Figure 8.

A complex exponential expression of beating signals is obtained from Equation (3) by Hilbert transform that can be written as:

(6) $$I(t)=2\sqrt{R(\tau )}E_{0}^{2}\exp[j2\pi (f_{0}\tau +f_{b}t-\frac{1}{2}\gamma \tau^{2})]S_{e}(t)S_{e}^{*}(t-\tau ),$$

where $S_{e}(t)=\exp[j2\pi e(t)]$ and $S_{e}(t-\tau )=\exp[j2\pi e(t-\tau )]$. The symbol * represents the complex conjugate. The nonlinear phase term e(t)−e(t−τ) is need to be eliminated. However, they cannot be eliminated directly because it is related to the time delay of the FUT. Whereas, the nonlinear phase term can be eliminated by the nonlinear phase of LO light e(t) and the nonlinear phase e(t−τ) of received light separately. The detail processing steps as follows:

*Step one:* $S_{e}(t)$ in Equation (6) can be restored by an auxiliary interferometer, LO light nonlinear phase e(t) being irrelevant to the length of FUT can be eliminated by a simple multiplication:

(7) $$I_{1}(t)=I(t)S_{e}^{*}(t)=2\sqrt{R(\tau )}E_{0}^{2}\exp\{j2\pi [f_{0}\tau +f_{b}t+\frac{1}{2}\gamma \tau^{2}-e(t-\tau )]\}.$$

From Equation (7), the received light’s nonlinear phase e(t−τ) is relevant to the time delay of the FUT. Many reflections occurs in the spatial domain including Rayleigh backscattering and Fresnel reflections, the received light’s nonlinear phase at the different time delay cannot be eliminated by a single reference function. To solve this issue, a time delay relevant time shift $\exp2\pi (j\pi f^{2}/\gamma )$ must be implemented to the beating signals by the deskew filter. By processing through the deskew filter, the received light’s nonlinear phase can be changed to time delay irrelevance.

*Step two:* The deskew filter is implemented to $I_{1}(t)$ in a frequency domain:

(8) $$I_{2}(t)=F^{-1}\{F\{I_{1}(t)\}\exp(j\pi f^{2}/\gamma )\},$$

where F and $F^{-1}$ signifies the Fourier transform and inverse Fourier transform, respectively. Inserting Equation (7) into (8), $I_{2}(t)$ can be given by:

(9) $$I_{2}(t)=2\sqrt{R(\tau )}E_{0}^{2}\exp\{j2\pi [f_{0}\tau +f_{b}t]\}F^{-1}\{S_{e}^{*}(-f)\exp(j\pi f^{2}/\gamma )\}(t),$$

where $F^{-1}\{\}(t)$ signifies an inverse Fourier transform with time variable *t* and $S_{e}^{*}(-f)$ is Fourier transform of $S_{e}^{*}(t)$. Here $S(t)=F^{-1}\{\exp(\pi f^{2}/\gamma )S_{e}^{*}(-f)\}(t)$, which is time delay irrelevance. 

*Step three:* The recovered beating signals $I_{3}(t)$ without any nonlinear phase can be calculated by removing S(t), i.e., multiplying with $S^{*}(t)$. S(t) can also be calculated by $S_{e}^{*}(t)$ processing through the deskew filter:

(10) $$S(t)=F^{-1}\text{\{}F\{S_{e}^{*}(t)\}\exp(\pi f^{2}/\gamma )\text{\}}=F^{-1}\text{\{}S_{e}^{*}(-f)\exp(j\pi f^{2}/\gamma )\text{\}},$$

and then:

(11) $$I_{3}(t)=I_{2}(t)\cdot S_{}^{*}(t)=2\sqrt{R(\tau )}E_{0}^{2}\exp\{j2\pi [f_{0}\tau +f_{b}t]\}.$$

**Figure 9:**
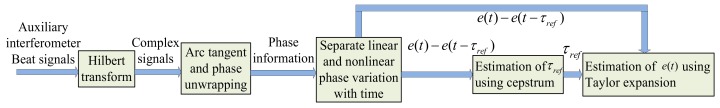
Signal processing procedure of nonlinear phase restoration from an auxiliary interferometer (reprinted from Ding et al. [34] with permission of SPIE; copyright (2014)).

The LO light’s nonlinear phase e(t) can be restored from an auxiliary interferometer. The steps of restoration of e(t) are illustrated in Figure 9. Du et al. utilize a cepstrum method to accurately restore the time delay in an auxiliary interferometer [24]. They also utilize the higher orders of Taylor expansion to restore the nonlinear phase with more “specific information” and reduce the errors due to the first order of Taylor expansion estimation.

**Figure 10:**
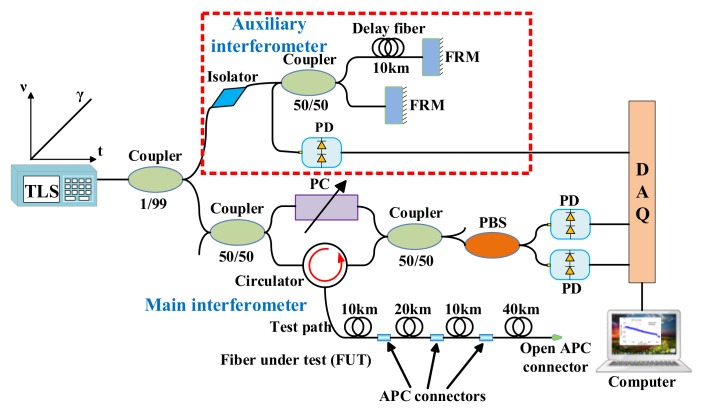
Experimental setup of the OFDR system using deskew filter method. TLS is tunable laser source. FRMs are Faraday rotating mirrors. PC is a polarization controller. PD is a photo-detector. PBS is a polarization beam splitter and DAQ is a data acquisition card (reprinted from Ding et al. [34] with permission of SPIE; copyright (2014)).

The experimental setup of deskew filter OFDR is shown in Figure 10. The main interferometer is a modified fiber-based Mach-Zehnder interferometer. The auxiliary interferometer is an unbalanced Michelson interferometer with a 10 km delay fiber. FUT is an 80 km SMF. Three APC-APC connections and one open APC connector are in FUT as shown in Figure 10. 

**Figure 11:**
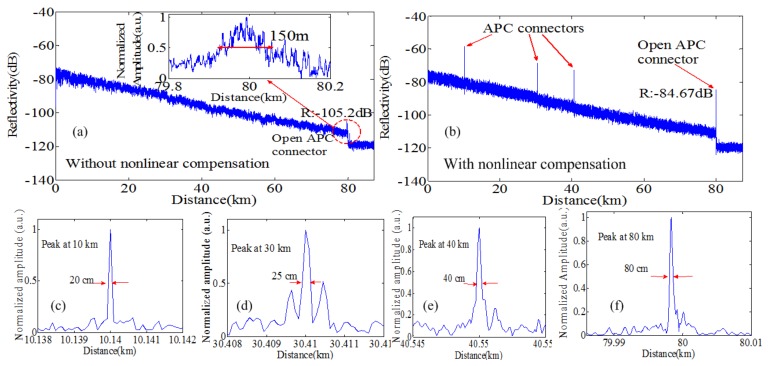
Measured Rayleigh backscattering and Fresnel reflections traces for an 80 km FUT with APC-APC connections and open APC connector without (**a**) and with (**b**) the nonlinear phase compensations. (**c**–**f**)’s y axis are transformed a logarithm to a normalized linear coordinate (reprinted from Ding et al. [34] with permission of SPIE; copyright (2014)).

They compared the experimental results of four Fresnel reflections profiles at locations of 10 km, 30 km, 40 km and 80 km with and without any nonlinearity compensation shown in Figure 11. The nonlinear phase compensation significantly improves the spatial resolutions of these reflections. The spatial resolution of Fresnel reflection at 80 km is 80 cm, which is enhanced by about 187 times than that by processing with a standard FFT. 

### 3.4. Short Tuning Range Method

As the phase noise will be aggravated along with a tuning range of TLS increasing, the sensing range can be increased by decreasing the tuning range. In addition, this relatively short tuning range of TLS restricts the spatial resolution of OFDR to few tens of meters but increases the tuning linearity of TLS during a tuning period [37]. 

#### 3.4.1. Narrow Linewidth Laser Method 

Geng et al. presented a 95 km OFDR system using a TLS with a linear tuning range of 380 MHz [15]. Ding et al. present a method to extend the measurement distance of a OFDR beyond the laser coherence length [16]. One hand, an optical frequency tuning range of TLS is decreased to be 53 MHz. On the other hand, the frequency tuning speed of TLS is increased to measure the refection phase noise term that contains the information of the reflectivity and position of the refection. 

**Figure 12:**
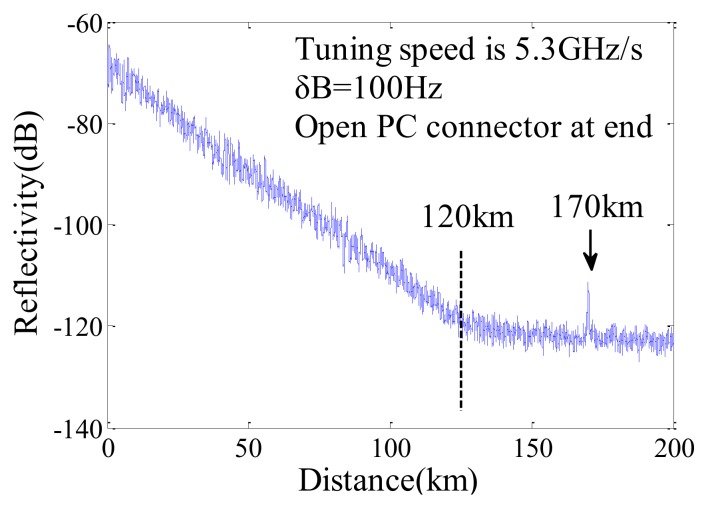
Measured Rayleigh backscattering and far-end Fresnel reflection trace from an open PC connector for 170 km. The range of Rayleigh backscattering is 120 km (reprinted from Ding et al. [16] with permission of IEEE; copyright (2013)).

Using a common OFDR with a TLS with a coherence length of only 13.6 km, the measurement distance of Fresnel reflection is up to 170 km and that of Rayleigh backscattering is up to 120 km shown in Figure 12. The noise floor is −120 dB and the dynamic range is ~53 dB.

#### 3.4.2. Dynamic OFDR

Arbel et al. presented a dynamic OFDR system which enables distributed acoustic sensing [37]. As this dynamic OFDR system operates with a fast tuning rate of TLS (~1012 Hz/s), the length of the time window recorded in every tuning range (tuning time) can be kept very short (16 μs). This ensures that acoustically induced phase variations during a tuning time remain small. The induced phase can be observed from one tuning time to the next. This presented OFDR can sense acoustic signals along the entire a 10 km FUT.

#### 3.4.3. Fractional Fourier Transform

Shiloh et al. used a Fractional Fourier Transform (FrFT) to analyze the output signals and acquire the backscattering traces. The method enables a high spatial resolution at 20 km sensing range with a high tuning rate of TLS. The spatial resolution of FBG’s-array is measured to be about 2.8 m using this system [38].

#### 3.4.4. Time-Gated Digital OFDR

**Figure 13:**
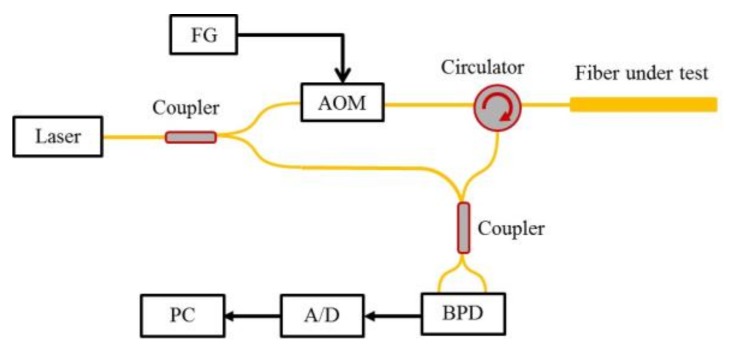
Experimental setup of the time-gated digital OFDR system. FG is function generator. AOM is acousto-optic modulator. BPD is balanced photo detector. A/D is analog-to-digital convertor. PC is personal computer (reprinted from Liu et al. [17] with permission of OSA; copyright (2015)).

Liu et al. presented a time-gated digital OFDR (TGD-OFDR) system. The input light is chirped and the chirp is gated within a narrow time window, namely the tuning range of TLS is very short [17]. The experimental setup of this TGD-OFDR is shown in Figure 13. The light from a TLS is split into two beams. The upper beam in the test path is a wavelength tuning and time-gated light modulating by an acousto-optic modulator (AOM), and the lower in the reference path is a wavelength-stable continuous light. A function generator (FG) with a chirp sinusoidal waveform is used to drive AOM. The input pulse in the test path has a short pulse duration, so the tuning rate of TLS can be set to very high independent on the length of FUT. 

**Figure 14:**
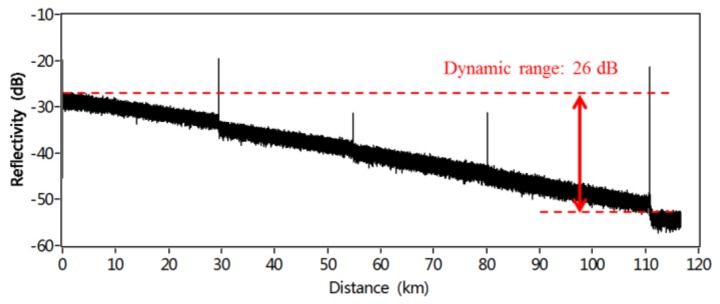
Measured trace of Rayleigh backscattering and Fresnel reflections along 110 km FUT (reprinted from Liu et al. [17] with permission of OSA; copyright (2015)).

**Figure 15:**
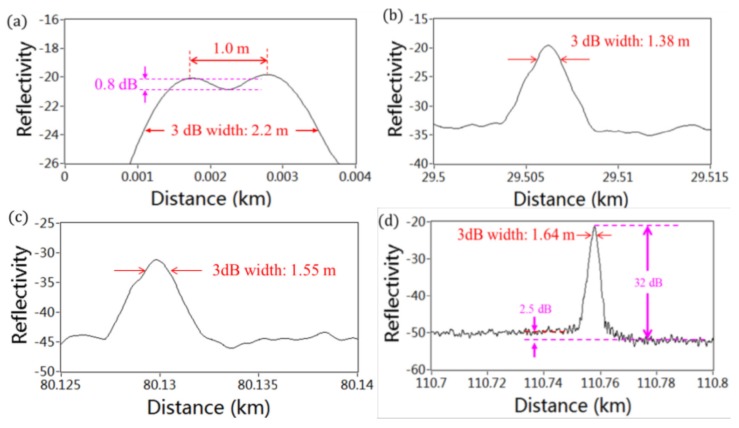
(**a**) Reflection profiles of the pigtail end. (**b**) APC-APC connection at 29.5 km. (**c**) APC-APC connection at 80.1 km. (**d**) Open PC connector at 110.7 km (reprinted from Liu et al. [17] with permission of OSA; copyright (2015)).

Figure 14 shows Rayleigh backscattering and Fresnel reflection trace along the FUT after averaging of 373 measurements. Five Fresnel reflections occur on the trace caused by the APC-APC connection and open PC connector. Figure 15a shows that two close Fresnel reflections due to the pigtail of a circulator can be distinguished by a pit of 0.8 dB depth. The spatial resolution at this position is measured to be 1.2 m. As shown in Figure 15b–d, the spatial resolutions are 1.38 m at 29 km, 1.55 m at 80 km, and 1.64 m at 110 km, respectively.

#### 3.4.5. Kerr Phase-Interrogator Based OFDR

Baker et al. presented an incoherent OFDR based on a Kerr phase-interrogator with a frequency-swept sinusoidal modulation [18]. This OFDR has a long sensing range beyond the laser coherence length. They experimentally demonstrate that the spatial resolution of Fresnel reflection points is about 11.2 cm at 151 km. As this type of OFDR belongs to an incoherent schematic [39], so it can only detect Fresnel reflections with strong reflectivity not Rayleigh backscattering.

### 3.5. Summary of Methods for Long Range in OFDR

Recently, the long range OFDR is a research hot spot, which is usually based on the last two types the compensation methods for the nonlinear phase noise in OFDR: one is the software algorithm and the other is the short tuning range method. A brief list of their performance summary is shown in Table 1.

## 4. Distributed Optical Fiber Sensing Based on OFDR

### 4.1. Principle of RBS Based Sensing

**Figure 16:**
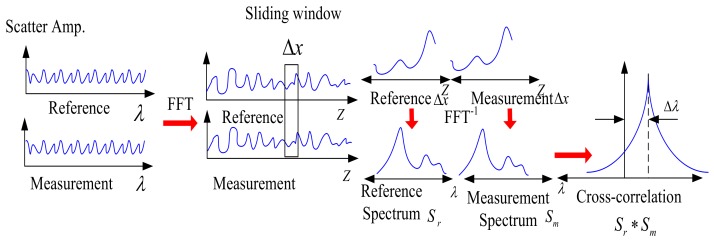
Signal processing procedure of RBS shifts based distributed sensing in OFDR.

OFDR can measure Rayleigh backscattering as distributed reflectivity patterns along a FUT length. Rayleigh scattering is caused by random refractive index fluctuations along a FUT, and it can be modeled as a long, weak FBG with random periods. Such a permittivity variation can be detected by the spectra of an beating interference between the test light from FUT and LO light [19,40]:

(12) $$\Delta \bar{\varepsilon }(x)=i\frac{n}{E_{0}^{2}rc\beta_{0}\pi }\int_{-\Delta \beta }^{\Delta \beta }I_{d}(\beta -\beta_{0})e^{-i\beta 2(x-x_{0})}d\beta$$

where *I_d_* is the measured spectra of the beating signal, *n* is the refractive index, *E*_0_ is the input optical field, *r* is the reflectivity of the LO light and *x*_0_ is the position of the LO light. The spatial domain signals contain phase and amplitude information in Equation (12), the spectra of any segment of FUT are converted by selecting the corresponding spatial domain signals and transformed it back to the optical frequency domain, namely the local RBS. The variations of the sensing parameters such as temperature or strain are measured by the spectral shifts in the local RBS. Froggatt et al. present a local RBS shifts based distributed strain and temperature sensing method. In this method, the sensing spatial resolution is up to 5 mm to 10 mm and the strain and temperature resolutions are 0.8 με and 0.08 °C, respectively [19,20]. The signal processing procedure of RBS shifts based sensing method in OFDR shown in Figure 16 is as follows [41]: 

(1) Operating OFDR system two times to acquire two signals in the optical frequency domain. One is considered as the reference signal and the other is considered as the measurement signal. Apparently, the two signals are acquired with the sensing parameters such as temperature or strain variations.

(2) Transforming the measurement and reference signals from the optical frequency domain to the spatial domain by a Fast Fourier transform (FFT).

(3) Dividing the total FUT to several segments using a sliding window with a ∆*X* length that contains *N* data points as the spatial local Rayleigh backscattering signals. ∆*X* is the effective sensing spatial resolution, which can be given by:

(13) ΔX=NΔZ,

where:

(14) ΔZ=c/2nΔν.

where ∆*Z* is the spatial resolution of one data point, *n* is the refractive index of FUT, and ∆ν is the optical frequency tuning range of the TLS.

(4) Padding *M* zeros for local Rayleigh backscattering segment in the spatial domain and the length for each segment is changed to be *M + N*.

(5) Transforming each local Rayleigh backscattering, by an inverse FFT back to the optical frequency domain, namely the local RBS.

(6) Operating the cross-correlation of the local measured RBS and referential RBS. The optical frequency shifts of the local RBS can be measured by the shifts of cross-correlation peak, which reflects the sensing parameters such as strain or temperature variation. The optical frequency measurement resolution in the system can be given by:

(15) $$\delta \nu_{\min}=\Delta \nu /M+N,$$

The minimal measurable sensing parameters variation *δS_min_* can be obtained with:

(16) $$\delta S_{\min}=\delta \nu_{\min}/RES=\Delta \nu /\left[RES\left(M+N\right)\right],$$

where *RES* is the ratio of the optical frequency shifts of the RBS and the sensing parameters variation, namely RI measurement sensitivity. 

Substituting Equations (13) and (14) into (16), we can obtain a clearer expression of the relationship of *δS*_min_ and ∆*X*:

(17) $$\delta S_{\min}=\frac{c}{2n\Delta X\times RES\times (M/N+1)}.$$

When we pad no zeros for local Rayleigh backscattering segment in the spatial domain as *M* = 0, Equation (17) can be given by:

(18) $$\delta S_{\min}=\frac{c}{2n\Delta X\times RES}.$$

Comparing with Equations (17) and (18), the value of *δS*_min_ is decreased by increasing the value of ∆*X* at the condition that RES is a constant based on Equation (18). Namely, if *δS*_min_ is improved, ∆*X* will be deteriorated (∆*X* increased) as shown in Equation (18) without any zero padding. When we pad zeros for the local Rayleigh backscattering segment in the spatial domain, the value of *δS*_min_ can be decreased without changing ∆*X* and *N* based on Equation (17). Namely, *δS*_min_ can be improved without sacrificing ∆*X*.

### 4.2. Strain Sensing

Froggatt et al. presented a local RBS spectral shifts based distributed strain and temperature sensing method with a high sensitivity and high spatial resolution [19,20]. The signal processing procedures of the RBS shifts based distributed strain sensing have been illustrated in the Section 4.1. 

LUNA Tech Inc. (Roanoke, VA, USA) has developed a commercial product of OFDR based on RBS shifts sensing method OBR 4600 [42]. The sensing spatial resolution is up to be ±1 cm. The strain resolution is up to be 1 με. The sensing range is about 70 m in a standard mode. It also extends the strain measurements up to a range of 2 km for applications located in a vibration environment.

### 4.3. Dynamic Strain

As the limitation of the tuning speed of TLS, with OFDR it is difficult to achieve a dynamic strain sensing. Some attempts have been made for this purpose. Zhou et al. measured a spectral shift of the local RBS continuously and achieve a dynamic strain sensing with a measurable vibration frequency 32 Hz. The spatial resolution is 10 cm and the sensing range is 17 m [43]. 

LUNA Tech Inc. has developed ODiSI-B 5.0, another commercial OFDR product which supports more dynamic testing environments. The frequency of ODiSI-B 5.0 can be more than 10 Hz in a sensing range of 2 m. The strain amplitude is ±600 με and the spatial resolution is up to be 5.2 mm. The strain accuracy is up to be ±15 με [44].

### 4.4. Temperature

The principle of distributed temperature sensing is similar to the strain sensing presented in the Section 4.1 and Section 4.2. Here we introduce some examples for the temperature sensing using OFDR in the harsh environments. Sang et al. presented a RBS shift based distributed temperature sensing of up to 850 °C in OFDR using a 1 m gold coating SMF [45].

Boyd et al. used a RBS shifts based distributed cryogenic temperature sensing method in OFDR to monitor the superconducting degaussing cables in cryogenic environments with the temperature being closed to 0 K. They obtain the temperature sensitivity curve that calculated form the calibration of the temperature and the RBS shift with the temperature ranges from 150 K down to 15 K [46,47].

### 4.5. Strain and Temperature Discrimination

One of the issues of the RBS shifts based distributed strain sensing in OFDR is that it has the cross-talk between temperature and strain. Undesirable RBS shifts caused by temperature could influence the strain sensing. Single measurement is difficult to distinguish between the RBS shifts caused by strain and temperature. The temperature and strain discrimination is very important to the RBS shifts based sensing. 

**Figure 17:**
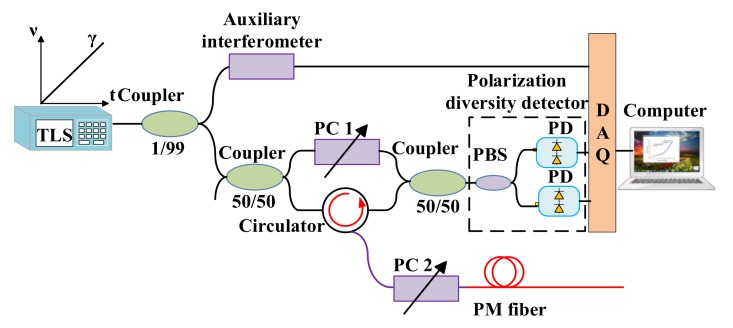
The experimental setup of OFDR for strain and temperature discrimination by PM fiber. TLS is tunable laser source. PC is a polarization controller. PD is a photo-detector. PBS is a polarization beam splitter and DAQ is a data acquisition card. Auxiliary interferometer provides the external clock for DAQ. This figure is adapted from Li et al. [50].

Froggatt et al. [48,49] and Li et al. [50] presented a strain and temperature discrimination method by autocorrelation and cross-correlation of RBS shifts in a polarization maintaining (PM) fiber. The experiment setup of OFDR for strain and temperature discrimination using PM fiber is shown in Figure 17. A polarization diversity detector includes two photo-detectors and a polarization beam splitter (PBS). Adjusting polarization controller (PC) 1 is to make “s” and “p” components of the light equal in the reference path. Adjusting PC 2 is to control the polarization states coupled to PM fiber and make the reflected light power of “s” and “p” components maximum. The autocorrelations of the RBS of the PM fiber are dependent on the temperature variation in PM fiber, whereas the cross-correlations of local RBS are dependent on both temperature and strain variations in PM fiber. The strain and temperature discrimination can be calculated by a parameter matrix of the temperature/strain coefficients in PM fiber [50]. 

Zhou et al. presented a temperature and strain discrimination method by combining Brillouin optical time-domain analysis (B-OTDA) and OFDR. However, this method is high cost and unpractical, because they need two complex setups B-OTDA and OFDR to realize this method [51].

**Figure 18:**
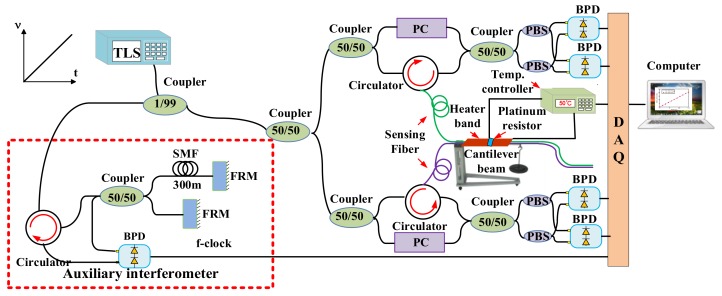
Experimental setup for distributed strain and temperature discrimination method using two types optical fiber in OFDR. TLS is tunable laser source. FRM is Faraday rotating mirror. PC is polarization controller. BPD is balanced photo-detector. DAQ is data acquisition card. The two types of sensing fiber standard SMF and RC SMF are paired side by side as FUT (reprinted from Ding et al. [52] with permission of IEEE; copyright (2016)).

Ding et al. presented a distributed strain and temperature discrimination method using two types optical fiber in OFDR [52]. Two types of SMF are paired side by side as FUT. One type is the standard SMF, and the other type is the reduced-cladding (RC) SMF. The experimental setup is shown in Figure 18. A heat band lays over FUT as a temperature excitation. The FUT is adhered on the cantilever beam by the glue. The weights are applied on the cantilever beam as a strain excitation. The platinum resistor is as the temperature sensor on the heat band and the temperature controller can acquire the temperature values and supply the electric current in the heat band. The strain on FUT can be calculated by the weights applied on the cantilever beam. As a results of the standard SMF and RC SMF have the disparate temperature and strain coefficients, the temperature and strain variation can be calculated simultaneously by the temperature and strain coefficients’ matrix.

### 4.6. Vibration

#### 4.6.1. CCSA Method for the Spatial Domain Signals

**Figure 19:**
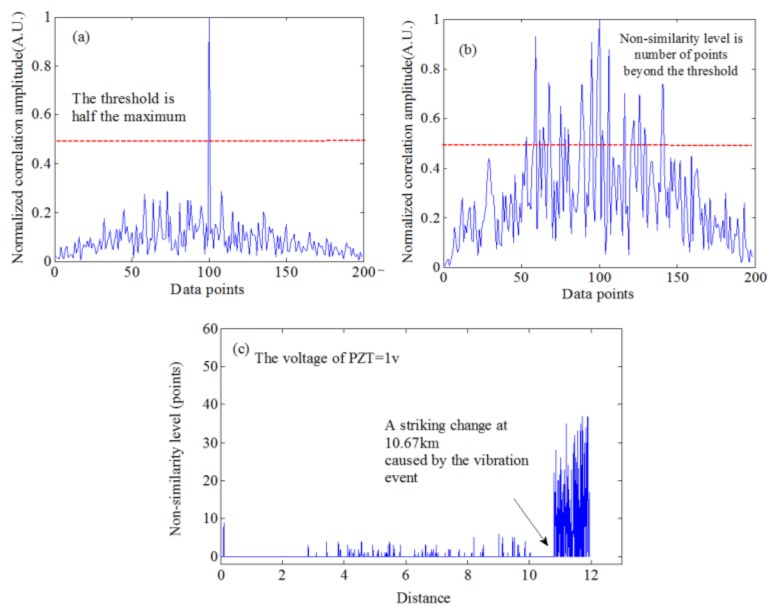
Cross-correlation between local Rayleigh backscattering in the spatial domain at the non-vibrated state and vibrated state: (**a**) not at the vibration location, (**b**) at the vibration location. A “non-similarity level” is number of points beyond a set threshold. (**c**) Measured distributed “non-similarity level” as a function of FUT length (reprinted from Ding et al. [53] with permission of OSA; copyright (2012)).

OFDR can also achieve a distributed optical fiber vibration sensing (DVOFS) with a long sensing range. Ding et al. presented a cross-correlation similarity analysis (CCSA) method using the spatial domain signals [53]. This frequency-sampling method is used to compensate the nonlinear phase in this OFDR system and the experimental setup is as similar as Figure 3. The length of delay fiber in the auxiliary interferometer is about 30 km. The length of FUT is about 12 km. Two piezoelectric transducers (PZT) fiber stretchers are installed to the FUT at locations of 10 km and 10.67 km, respectively, to serve as two vibration events. Rayleigh backscattering can be acted as a FUT “fingerprint”. The vibrations on an FUT give rise to a variation in the “fingerprint” of FUT, which can be utilized to detect vibration events on FUT. The similarity of local spatial Rayleigh backscattering signals is analyzed by a cross-correlation of two separated measured data. When the “similarity” of “fingerprint” on FUT is high, the cross-correlation results have one center peak, whereas, when a “similarity” of “fingerprint” on FUT is low, the cross-correlation results are disordered, the center peak is low and it occurs many disordered peaks spreading along the center peak shown in Figure 19a,b. A variation in the “non-similarity level” is utilized to locate a vibration event. The distributed “non-similarity level” data along the FUT is shown in Figure 19c. In addition, the vibration frequency is extracted from the Bessel function term in the spatial domain signals. The maximal measurable frequency of vibration events can be increased up to 2 kHz or even higher with a sensing spatial resolution of 5 m. This system can sense two concurrent vibration events at a sensing range of 12 km. Moreover, this system can achieve two vibrations located at different positions of FUT. 

#### 4.6.2. CCSA Method for the Optical Frequency Signals

**Figure 20:**
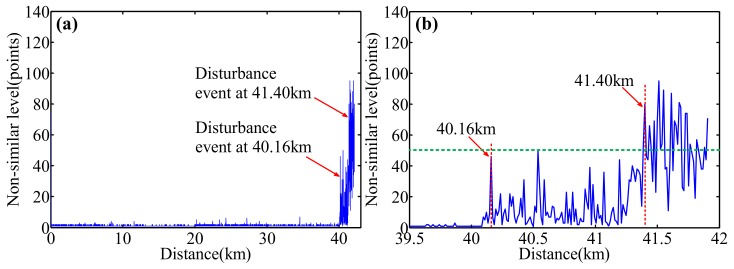
Measured distributed “non-similarity level” as a function of a FUT length with two concurrent vibration events. The PZT 1 at 40.16 km is applied by a triangle wave with 2 V. The PZT 2 at 41.40 km is applied by a triangle wave with (**a**) 2 V, (**b**) 3 V (reprinted from Ding et al. [54] with permission of IEEE; copyright (2016)).

Based on the CCSA method for the spatial domain signals, an optimized deskew filter method [21,24] is used to compensate the nonlinear phase in OFDR and extend the sensing range. The experiment setup is similar to the one in Figure 4 and Figure 10. The length of FUT is about 42 km. Two PZTs are added at the positions of 40.16 km and 41.40 km on FUT, respectively, to serve as two vibration events. They acquire the location and level information of the vibration events at 40 km with a sensing spatial resolution of 11.6 m by a cross-correlation of local RBS between the non-vibrated and vibrated signals. Comparing with the CSSA method for spatial domain signals [53], in the CSSA method for optical frequency domain signals [54], as the local Rayleigh backscattering signals in the spatial domain are converted to the optical frequency domain, the convolution noise could be converted to the multiplicative noise, which makes characteristic information in optical frequency domain more robust than in the spatial domain. The experimental results are shown in Figure 20a,b. 

Two vibration events can be located by the “non-similarity level” of two local RBS between vibrated and non-vibrated states. Although the first vibration event makes the all “non-similar levels” high after this point, the second “step” variation is greater than that of the first one as a result of a superposition from the two vibration events, i.e., a superposition level from two vibration events. Therefore, these two concurrent vibration events can be located by the “step” variation of “non-similar levels” of all FUT.

#### 4.6.3. M-CCSA Method 

**Figure 21:**
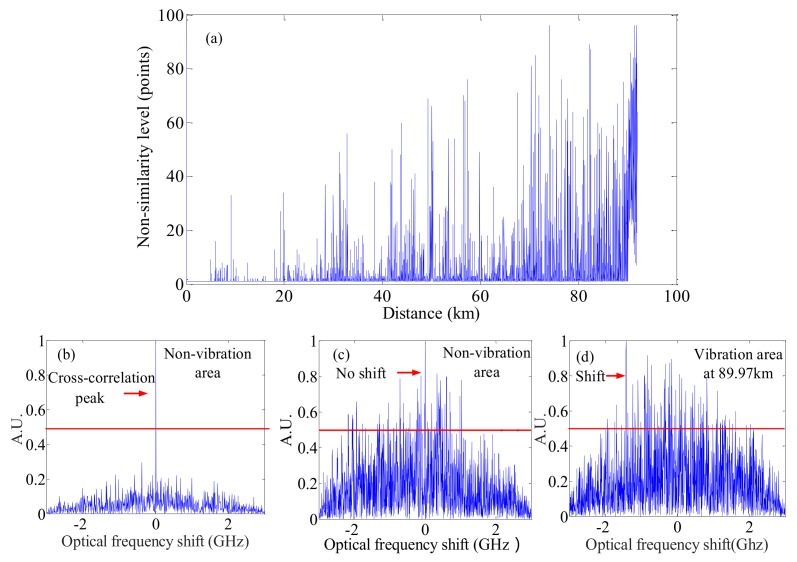
(**a**) Measured distributed “non-similarity levels” as a function of FUT length. Vibration events cannot be located by significant variations of “non-similarity level”. (**b**) Cross-correlation between two local RBS in the non-vibration area at a short sensing range, (**c**) in the non-vibration area at a long sensing range, (**d**) in the vibration area (89.97 km). The red line is half of the cross-correlation peak and the cross-correlation peak is indicated by the red arrow (reprinted from Ding et al. [55] with permission of IEEE; copyright (2017)).

**Figure 22:**
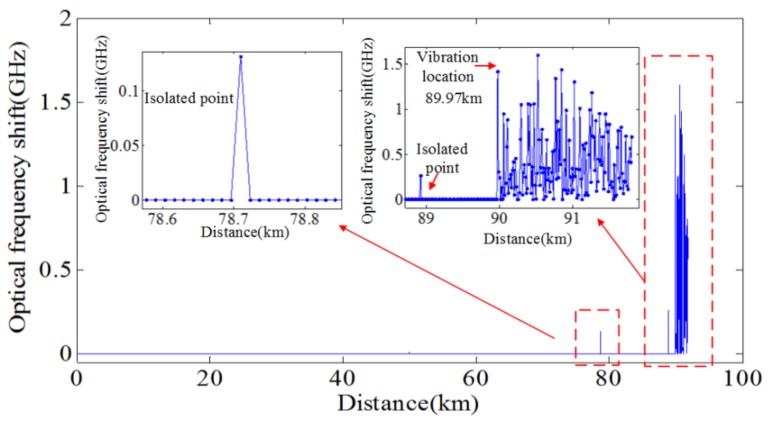
Measured distributed local RBS shifts as a function of FUT length (reprinted from Ding et al. [55] with permission of IEEE; copyright (2017)).

To lengthen the sensing range of OFDR based DVOFS, Ding et al. presented a long-range OFDR- based DVOFS with multi-characteristics of Rayleigh backscattering, namely the M-CCSA method [55]. The experimental setup is as similar to that shown in Figure 4 and Figure 10. The length of FUT is about 92 km. Two PZTs are added at the positions of 89.97 km and 91.23 km on FUT. The original CCSA method based on single characteristic of Rayleigh backscattering [54] is applied to process the local RBS for each section of FUT to acquire distributed “non-similarity levels” shown in Figure 21a. In the original CCSA method [54], the location of the “step” variation of the “non-similarity levels” along the FUT is where the vibration event occurs. However, from the 90 km data processing though by the original CSSA method, the cross-correlation results show one center peak in the non-vibrated area at a short measurement distance (<5 km) shown in Figure 21b. However, as the sensing range increasing, the cross-correlation results show disordered and the “non-similarity level” is high, even if the sections of FUT locate at the non-vibrated area shown in Figure 21c. The distributed “non-similarity levels” as a function of distance are disordered shown in Figure 21a, so the vibration events cannot be located by the original CCSA method [54]. The spectral shift of local RBS in the optical frequency domain, as an alternative characteristic of Rayleigh backscattering is applied to process the same data. The distributed local RBS spectral shifts along FUT are shown in Figure 22. The local RBS spectral shift is zero at the non-vibrated area. The first “step” variation at 89.97 km in Figure 22 can be used to locate the first vibration event. The first vibration event is detected and located by the local RBS shifts considered as an alternative characteristic of Rayleigh backscattering. However, the RBS shifts based method cannot locate the second vibration event. 

**Figure 23:**
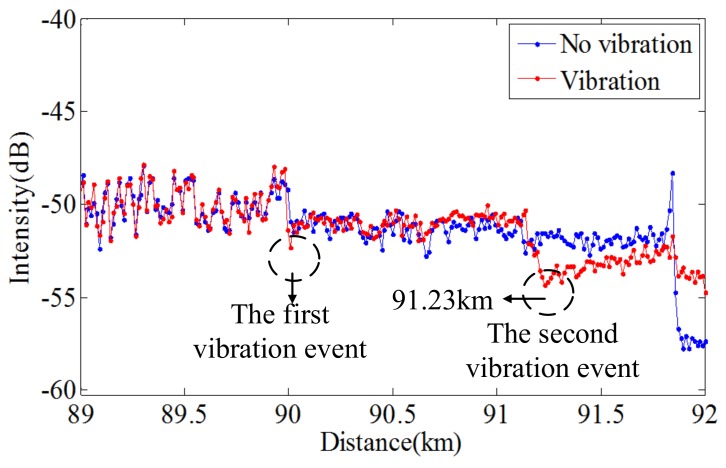
Measured spatial Rayleigh backscattering trace. An obvious “V” shape characteristic at 91.23 km can be used to located the second vibration event (reprinted from Ding et al. [55] with permission of IEEE; copyright (2017)).

Beside the local RBS spectral shift considered as Rayleigh backscattering characteristic information, more alternative Rayleigh backscattering characteristic need to be excavated to sense and locate the second vibration event. As the vibration on FUT could result in many Bessel function terms in the beating signals, so more power of the spatial Rayleigh backscattering will spread to the adjacent lobes [53]. From the spatial OFDR signals shown in Figure 23, using “V” shape characteristic is difficult to locate first vibration event (about 90 km), as “V” shape is not very obvious due to the stochastic fluctuation of Rayleigh backscattering. However, the “V” shape caused by the second vibration event is pronounced as a result of the cumulative effect of the two vibration events. The “V” shape characteristic can be used to sense and locate the second vibration event. The lowest position of the “V” shape is where the second event occurs. Above all, OFDR based DVOFS by multi-characteristics of Rayleigh backscattering can sense two concurrent vibration events. The sensing range is about 92 km and the vibration event locating accuracy (spatial resolution) is about 13 m.

#### 4.6.4. Short Tuning Range Method 

The CSSA method [54] and M-CSSA [55] method cannot measure the frequency of the vibration event. OFDR with short tuning range has a capability of measuring the vibration frequency. The tuning range of this type OFDR is about several megahertz to several ten megahertz. As TLS operates with a high tuning speed, the tuning period can be kept rather short (several micro-seconds) to realize a high time resolution for measuring high vibration frequency. The short tuning range can also ensure that the phase variations during a single tuning period remain small. The acoustically induced phase variation can be detected from one scan to the next. The core idea of OFDR with short tuning range is to implement a linearly tuning pulse as an input light, which can be considered as a combination of OFDR and phase sensitive OTDR.

**Figure 24:**
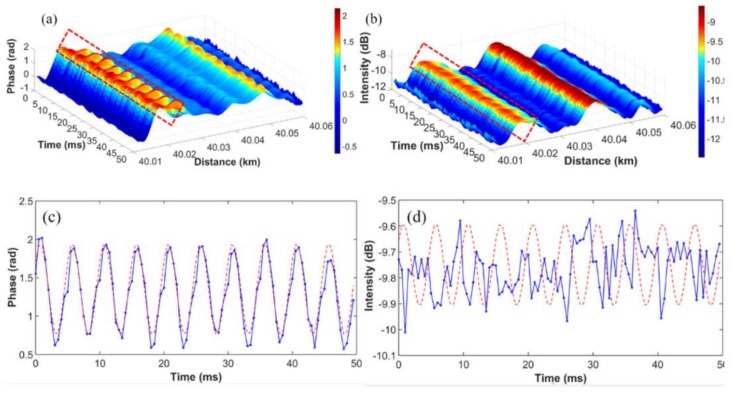
Experimental results of DVOFS based on TGD-OFDR with a 200 Hz sinusoidal excitation at z = 40.02 km. (**a**) Distance-time mapping trace of the phase term of Rayleigh backscattering. (**b**) Distance-time mapping trace of the intensity term of Rayleigh backscattering. (**c**) Phase-time curve of a vibration event. (**d**) Extracted intensity-time curve of a vibration event (reprinted from Wang et al. [56] with permission of OSA; copyright (2015)).

Wang et al. presented a DVOFS based on phase extraction from time-gated digital OFDR (TGD-OFDR) [56]. The experimental setup of TGD-OFDR is shown in Figure 13, which has been illustrated in the Section 3.4.4. A 90 degree optical hybrid is utilized to extract phase information. By increasing the tuning rate of TLS, the influence of environmental acoustically induced phase on TGD-OFDR is reduced greatly, which makes the phase extraction in TGD-OFDR more reliable than that of the conventional OFDR, leading to realize a quantitative vibration measurement at a long sensing range. As shown in Figure 24, this system can measure a vibration event with the frequency of 200 Hz and a minimum acceleration speed of 0.08 g. In Figure 24a–d, the distance-time mapping of the phase term has a much better sensitivity than that of the amplitude term. By this presented method, the sensing range of DVOFS based on TGD-OFDR is up to 40 km, the measurable frequency is up to 600 Hz and the minimal vibration acceleration speed is about 0.08 g.

Steinberg et al. used a similar principle as [56] to realize a dynamic ultra-sensitive DVOFS with a sensing range of 101 km and acoustical sampling rate of 600 Hz [57]. They use this system to detect and record drops of two small paperclips (~1 g) from height of ~20 cm on two fiber segments of FUT as two vibration events. Two fiber sections are 10 m apart at the end of the 101 km fiber. Two vibration events induced signals are high SNR and don’t have serious cross-talk between two vibration events.

#### 4.6.5. Summary of DVOFS Based on OFDR 

To illuminate the features of different methods in the DVOFS based on OFDR clearly, we give a brief list of their performances summary in Table 2.

### 4.7. Pressure 

The transversal pressure sensitivity of the RBS shifts based distributed sensing method in OFDR is so small that makes the direct pressure measurement not feasible, which is similar to FBGs based sensing methods. Thus, the transversal pressure is need to convert to longitudinal strain exerted on the FUT. To achieve this purpose, the materials with high Poisson’s ratio and low Young modulus i.e., polymer are used to package FUT. A pressure applied to the packaging materials is converted to a longitudinal strain according to packaging materials’ Poisson’s ratio [58]. 

**Figure 25:**
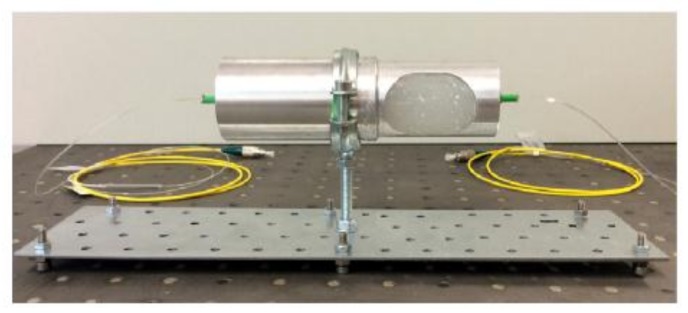
Prototype of simultaneous measurement of hydrostatic pressure and temperature based on polymer cylinder filling in a dual chamber system (reprinted from Schenato et al. [58] with permission of Elsevier; copyright (2016)).

Schenato et al. presented an RBS shifts based sensor for a concurrent measurement of hydrostatic pressure and temperature [58]. The proposed sensor consists of two chambers shown in Figure 25. One chamber filling polymer cylinder is used to convert the transversal pressure to longitudinal strain exerted on the embedded FUT. Another empty chamber is used to temperature measurement. The temperature and pressure sensitivities of the proposed sensor are approximately 61,702 GHz/°C and 613.202 GHz/kPa, respectively. The temperature and pressure measurement accuracies are 0.502 °C and 0.302 kPa, respectively.

**Figure 26:**
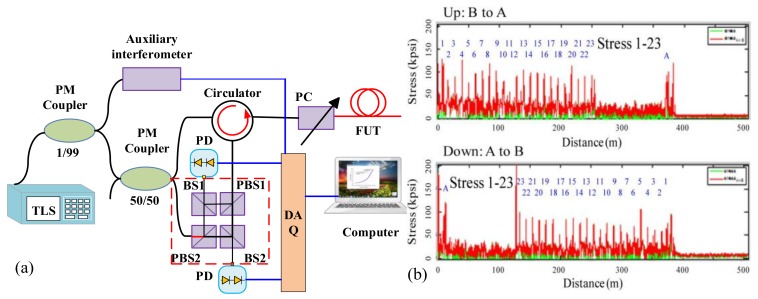
(**a**) Experimental setup of distributed transversal stress sensing by the local birefringence information using a P-OFDR. TLS is tunable laser source. PC is a polarization controller. PBS is polarization beam splitter. PD are photo-detectors. BS 1 and 2 are free-space polarization insensitive beam splitters. DAQ is data acquisition card. Auxiliary interferometer provides the external clock for DAQ. This figure is adapted from Wei et al. [59]. (**b**) Distributed stress measurement of a 250 m fiber coil (reprinted from Wei et al. [59] with permission of OSA; copyright (2016)).

Beside the RBS shifts based distributed pressure sensing, the transversal pressure can be measured by the birefringence in FUT. Wei et al. presented a polarimetric optical frequency domain reflectometer (P-OFDR), which can measure the transverse pressure from the birefringence information carried from Rayleigh backscattering on a 800 m FUT length with a spatial resolution of 0.5 mm [59]. The pressure ranges from 10 kpsi (69 Mpa) to 200 kpsi (1379 Mpa) and the measurement uncertainty is about 10%. The experimental setup is shown in Figure 26a. A polarization controller is used to vary the state of polarization (SOP) before entering and after exiting the FUT to evenly cover the Poincaré sphere [60]. The Rayleigh backscattering from different locations of FUT interfere with the light from TLS by a polarization diversity interferometer shown in the dotted-line box of Figure 26a. The interference signals are detected by PDs, and the local birefringence information along FUT can be then calculated by distributed SOP on FUT. The distributed transverse stress can be calculated by the local birefringence information along FUT. Figure 26b shows an example for distributed stress sensing for the fiber coils in fiber-optic gyroscopes by P-OFDR which can be utilized to decrease the stresses in a winding process and improve the quality of fiber coils. 

### 4.8. 3D Shape Sensing

3D shape sensing is an important and hotspot research direction in the optical fiber sensing. The RBS shifts based distributed strain sensing is an ideal technology for a 3D shape sensing. Duncan et al. present a 3D shape sensing by the RBS shifts based distributed strain sensing [61,62]. In 3D shape sensing, three fibers or multi-core fiber are comprised to a fiber triplet. The shape of the fiber triplet is reconstructed using the distributed strain values detected by OFDR. The fiber triplet is divided into many sections and evaluate the location of each section in its own frame. Then, a geometrical assumption is used to find the angle between the axis and the rotational axis of the segment along the fiber triplet. Parent et al. used this method to enhance the precision of surgical needle shape tracking [63]. The practical application of 3D shape sensing can be found in Ref. [64]. 

### 4.9. Magnetic Field

**Figure 27:**
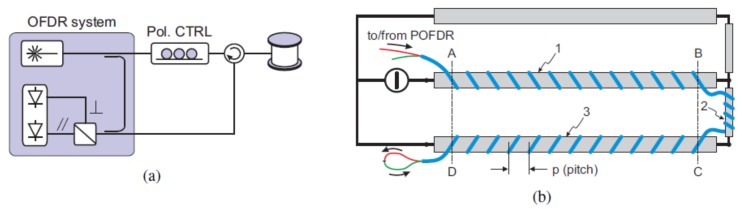
(**a**) Experimental setup of P-OFDR. (**b**) Experimental setup of the loose tube helically wound around conductors No. 1, 2 and 3 contains FUT, two of which are concatenated (drawing not in scale) (reprinted from Palmieri et al. [65] with permission of OSA; copyright (2015)).

**Figure 28:**
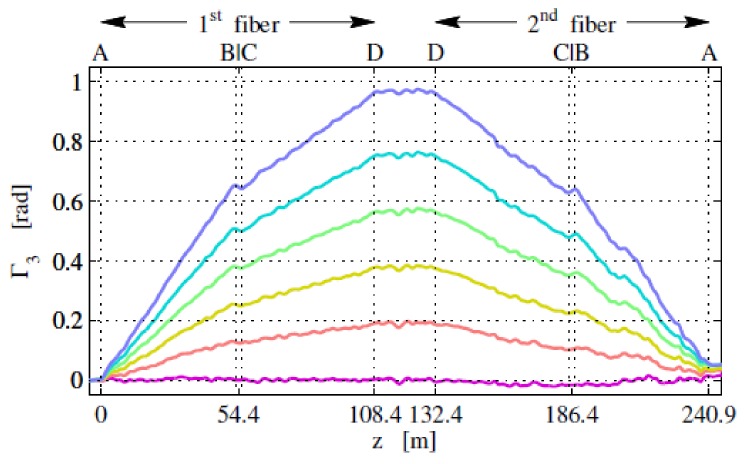
Accumulated Faraday rotation for different current intensities (reprinted from Palmieri et al. [65] with permission of OSA; copyright (2015)).

Palmieri et al. firstly used the P-OFDR method to realize a distributed sensing of electric current [65]. P-OFDR measure distributed Faraday rotation carried in Rayleigh backscattering of a 400 m FUT length with a spatial resolution of 4 m. The measurable current is up to 2.5 kA and the resolution is about 100 A. P-OFDR experimental setup is shown in Figure 27a, which consists of a standard OFDR with a polarization controller in the test path, utilizes to adjust the input SOP. The polarization diversity photo-detectors are used to analyze Faraday rotation carried in local Rayleigh backscattering. The experimental setup of the electric current sensing is shown in Figure 27b. The electric circuit is fabricated by three parallel conductors with a length of 20 m, 1.2 m apart, and connected in such a way that the current flowing in the central conductor is split in half in each of the outer ones. Figure 28 shows the P-OFDR measurement results of Faraday effects induced phase variations in different current intensities.

**Figure 29:**
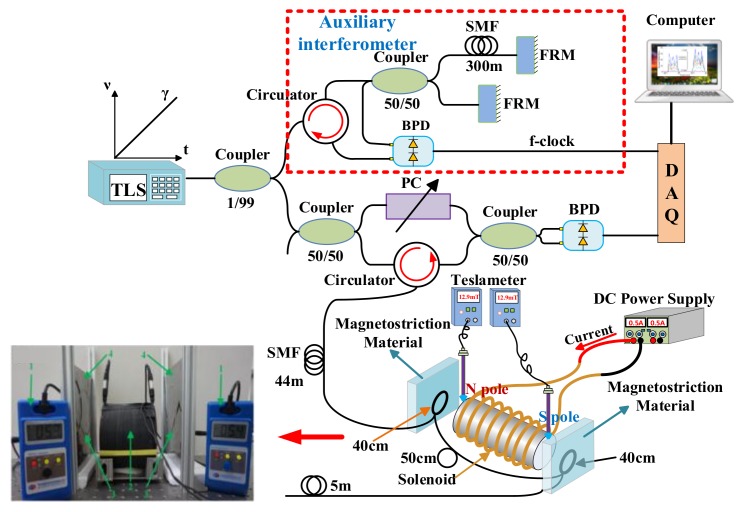
Experimental setup for distributed magnetic field measurement. TLS is tunable laser source. FRM is Faraday rotating mirror. PC is polarization controller. BPD is balanced photo detector. DAQ is data acquisition card. The photograph of magnetic field measurement setup. 1: Teslameter, 2: iron-core solenoid, 3: sensing fiber, 4: Fe-Co-V alloy thin films (reprinted from Ding et al. [66] with permission of The Japan Society of Applied Physics; copyright (2015)).

In addition, Du et al. presented a distributed optical-fiber magnetic-field sensor based on magnetostriction using RBS shift in OFDR [66]. The standard OFDR experimental setup is shown in Figure 29. The 51 m SMF as FUT is spliced with magnetostrictive Fe–Co–V alloy thin films. OFDR measure the strain on FUT caused by the magnetic field and then acquire the intensity of the magnetic field. The intensity of the magnetic field ranges from 0 to 143.3 mT. The minimum variation of the measurable magnetic intensity is 12.9 mT when the sensing spatial resolution is 4 cm, and it can be improved to 5.3 mT by increasing the sensing spatial resolution to 14 cm. This experimental setup also detects electric currents ranging from 0 to 11.8 A and 0 to 12.14 A at two positions along with a distributed current trace [67]. The minimal variation of the measurable electric current is 0.3 A with a sensing spatial resolution of 14 cm.

### 4.10. Refractive Index

**Figure 30:**
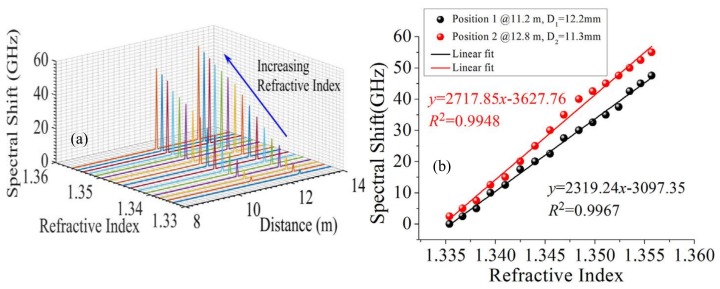
(**a**) Relationship between local RBS shifts and distance at various RIs. The locations of two macro-bending SMF are 12.2 mm and 11.3 mm, respectively. (**b**) The RI measurement sensitivities curves at location of 11.2 m and 12.8 m (Reprinted from Du et al. [68] with permission of Elsevier; copyright (2017)).

Du et al. presented a distributed refractive index (RI) sensor based on RBS shifts using macro-bending SMF in OFDR [68]. FUT is fabricated by bending a piece of SMF to a radius of curvature in several millimeters. The RI variation of the external medium surrounding the macro-bending fiber could cause local RBS shifts. RI ranges from 1.3348 to 1.3557 using this proposed method. This sensor also can measure multipoint RI variations simultaneously. Figure 30a shows the relationship between local RBS shifts and distance at various RIs. The RBS shifts only occur at the locations of macro-bending, so there is no any cross-talk from other fiber regions without bending structures. The RI measurement sensitivities are 2319.2402 GHz/RIU (18.5502 nm/RIU) and 2717.8502 GHz/RIU (21.7402 nm/RIU) with bending diameters of 12.202 mm and 11.302 mm, respectively shown in Figure 30b.

### 4.11. Radiation

**Figure 31:**
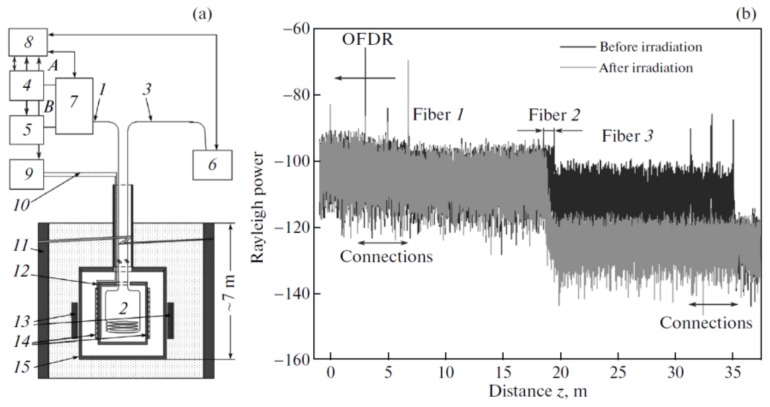
(**a**) Design of the experimental setup. (1–3) is Optical fibers, Fiber 1 and 3 are standard SMF. Fiber 2 is radiation sensitive fiber. (4) OFDR. (5) Superluminescent source. (6) Spectrum analyzer. (7) Fiber switch. (8) Computer. (9) Temperature controller. (10) Thermocouple element. (11) Water tank. (12) Furnace (13) 60 Co sources. (14) Heating elements. (15) Irradiation container. (**b**) Rayleigh backscattering trace before (black curve) and after (gray curve) irradiation (reprinted from Faustov et al. [69]. with permission of Springer Nature; copyright (2015)).

Faustov et al. presented a distributed measurement of absorbed γ radiation doses using OFDR [69]. The measurement range is up to 100 kGy and the relative accuracy is about 20%. The industrial test setup is shown in Figure 31a. The measurement principle is based on the detection of radiation induced absorption. The phosphor–silicate optical fiber is used as FUT, because it is radiation sensitive fiber with an increased sensitivity to irradiation [70,71]. OFDR detects a radiation by measuring the loss of Rayleigh backscattering. Figure 31b shows Rayleigh backscattering trace with a spatial resolution of 38 m before and immediately after irradiation. A loss “step” caused by the radiation induced absorption in radiation sensitive fiber (fiber 2) can be obviously detected from the OFDR trace at the locations of 18.6 to 19.2 m. 

### 4.12. Gas

**Figure 32:**
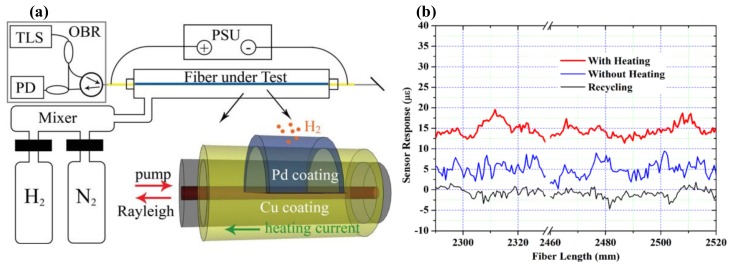
(**a**) Experimental setup of the distributed hydrogen sensing system using electrically heated optical fiber. OBR is optical backscatter reflectometer, namely OFDR. TLS is tunable laser source. PD is photo-detector. PSU is power supply. (**b**) The sensor strain response to hydrogen exposure at different positions on the same fiber (reprinted from Chen et al. [72]. with permission of AIP Publishing LLC; copyright (2012)).

Chen et al. presented a distributed hydrogen sensing method using RBS shifts in OFDR. The FUT is a special fiber with coating copper (Cu) and palladium (Pd) [72]. Pd hydrogen absorptions can give rise to a local strain variation that can be spatially measured by RBS shifts in OFDR. The hydrogen response and the sensor recycling can be accelerated by heating the Pd coating using the electrical power. The experimental setup of distributed hydrogen sensing is shown in Figure 32a. 

The 2 m long SMF as FUT is uniformly coated with 20 μm thick Cu alloy and further sputter-coated with 1 μm thick palladium on one side of FUT surface. A 350 mm long Pd/Cu coated FUT is installed into the gas chamber and sealed with fiber ferrules at both ends of FUT. 10% concentration hydrogen with nitrogen gas are sent into the chamber. The FUT can be heated by a current supply. The results are shown in Figure 32b. The strain are generated after the hydrogen exposure and shut-down of the on-fiber heating by OFDR. The heated sensor response is about three times larger than the unheated response. This method achieves a distributed hydrogen leak sensing with a spatial resolution of 1 cm.

### 4.13. Flow Rate

**Figure 33:**
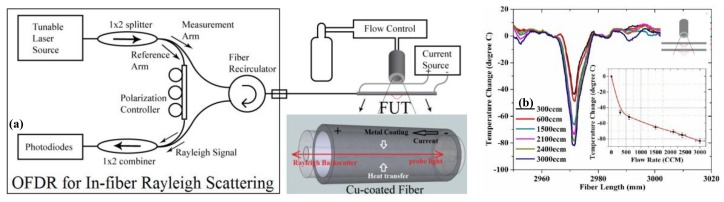
(**a**) Experimental setup of distributed hot-wire flow sensing using OFDR. (**b**) (Color Online) A local flow-induced temperature variation and flow rate sensitivity curve (reprinted from Chen et al. [73] with permission of OSA; copyright (2012)).

Chen et al. presented a distributed hot-wire flow sensing using a single piece of self-heated optical fiber in OFDR [73]. The experimental setup is shown in Figure 33a. SMF with 9 μm diameter Ge-doped core and 125 μm cladding is used as FUT and it is coated with 20 μm thick of copper alloy. The metal surface of FUT can be heated by an electrical current. The gas flows blowing on a segment of the FUT that cause a local temperature variation. OFDR can detect the magnitude and location of flow-induced temperature variation by RBS shifts. Figure 33b shows the temperature variation of the front-line hot optical wire to 300–3000 ccm (cubic centimeter per minute) gas flows blown from a quarter inch nozzle 5 mm away. This proposed sensor can detect the flow rate, location, and flow direction, simultaneously. 

### 4.14. Special Fiber

**Figure 34:**
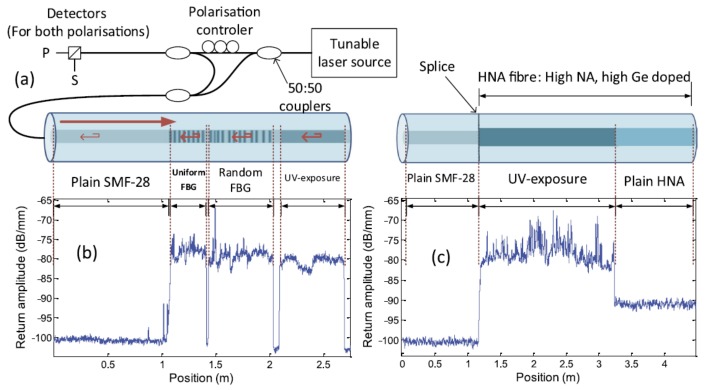
(**a**) OFDR system. (**b**) A comparison of various types of structure and exposure in a standard SMF-28 fiber: uniform FBG, random FBG and continuous UV exposure (no FBG). (**c**) A comparison of SMF-28, with UV-exposed and un-exposed HNA fiber (HNA: High NA fiber with high Ge core) (reprinted from Loranger et al. [40] with permission of Springer Nature; copyright (2015)).

The Rayleigh backscattering of SMF for optics telecommunication becomes lower and lower, because the impurities in SMF are less and less. The main limitation in the sensitivity and accuracy of distributed sensing based on RBS shifts in OFDR comes from the low Rayleigh backscattering signals in optical fiber. Rayleigh scattering enhanced fiber is a potential way to increase the sensitivity and accuracy in the RBS shifts based sensing methods. Loranger et al. presented a method to improve temperature and strain measurement sensitivity by an order of magnitude using a Rayleigh scattering enhanced fiber [40]. Rayleigh scattering enhanced fiber is fabricated by simply exposing the fiber core to UV light, which creates a high density of scattering defects. This process can be finished by any UV laser (solid state, argon) without any critical alignment or vibration control unlike writing FBGs. The OFDR system is shown in Figure 34a. Figure 34b shows that a continuous UV exposure generates a 20 dB enhancement in Rayleigh backscattering of FUT. Figure 34c shows that the UV-exposed a photosensitive high Ge doped core fiber (HNA fiber) has a same improvement in Rayleigh backscattering, comparing with that of UV-exposed standard SMF (i.e., SMF-28). Using this Rayleigh scattering enhanced fiber, they achieve a 20 mK temperature detection with a spatial resolution of 2 cm using RBS shifts based sensing in OFDR. In addition, Yan et al. used a femtosecond ultrafast laser to fabricate Rayleigh scattering enhanced fiber that are stable at high temperatures [74]. Rayleigh scattering enhanced fiber is fabricated by utilizing ultrafast laser pulses to scan the fiber cores continuously. This fabrication processing don’t need control the precision of grating periods at an order of nanometer like producing FBGs. Using this Rayleigh scattering enhanced fiber, they can realize a distributed temperature sensing with a spatial resolution of 5 mm at 800 °C using a RBS shifts-based method in OFDR.

Polymer optical fibers (POF) can also be used as a sensing medium for OFDR sensing. POF has low cost, good flexibility, enabling ultra-small bending radius, so POF based sensor have better mechanical than silica-based optical fibers. POF is one of the promising candidates for shape sensing, RI sensing and so on. Kreger et al. used POF to achieve distributed strain and temperature sensing in a range of 70 m. The sensitivity is roughly 20% higher than that of using silica-based optical fibers [75]. 

The photonics crystal fibers (PCF) or microstructured optical fibers (MOF) have some special features compared with standard telecommunications optical fiber, which can be used for novel sensing applications. For example, as PCFs have a lower radiation-induced attenuation than the standard optical fiber, they can be used to OFDR based temperature sensing in environment with X-ray irradiation [76]. In addition, the PCF is also a potential sensing medium for chemical and biological sensing. PCF have been used in single-point sensors for gas sensing [77] and DNA detection [78]. PCF as FUT that are very promising for distributed chemical and biological sensing with OFDR in the future.

## 5. Conclusions

We have presented a comprehensive and systematic overview of the principles and key technologies of OFDR, especially the sensing mechanisms and the applications of distributed optical fiber sensing based on OFDR reported in recent times. To summarize we list some perspectives of distributed optical fiber sensing based on OFDR:

First, in an OFDR system, TLS is a key device for the OFDR performance. The narrower width and high tuning linearity of TLS will increase the performance of the OFDR such as the measurement range, the spatial resolution and SNR, etc. In addition, TLS with a high tuning rate is an important way to achieve dynamic measurements and 3D shape sensing. The methods of nonlinear phase restoration in OFDR signals will also need to be explored. Some novel signal processing algorithms will be applied to solve this issue.

Second, OFDR can achieve a better sensing performance in OFDR-based sensing than before. In the distributed vibration sensing, OFDR-based technologies have a longer sensing range as well as a higher spatial resolution than that of OTDR-based technologies. Along with the developments of TLS with a high tuning rate, the dynamic measurements for strain and temperature with a high spatial resolution will be developed further. OFDR is a promising candidate for 3D shape sensing. With improvements of OFDR demodulation speed and implementation of Rayleigh scattering enhanced fiber, OFDR systems can achieve a real-time 3D curve shape reconstruction, which will be very useful for medicine, mechanical automation, aerospace structure monitoring, etc. Lastly, OFDR will extend to broader sensing parameters than before. OFDR theoretically can replace a lot of FBG-based sensing applications. Special fibers as a sensing medium for OFDR will become a hotspot in OFDR. Rayleigh scattering enhanced fibers, POF, PCF, fibers with a special coating and other types of special fiber will can sense more parameters than possible only using a standard SMF. The sensing parameters of OFDR can sense not only physical parameters, but also the chemical and biological parameters. As OFDR has a characteristic of a distributed sensing with a high spatial resolution, that could solve challenges that cannot be addressed by traditional single-point sensors. 

## Figures and Tables

**Table 1:** Performance summary of software algorithms and short tuning range methods for compensation of the nonlinear phase noise in OFDR.

Type	Method	Author	Sensing Range	Spatial Resolution
	NUFFT [29]	Ding et al.	51 m	5 cm
Software algorithm	Cubic spline interpolation [27]	Song et al.	300 m	0.3 mm (theoretical value)
	Concatenately generated phase [33]	Fumihiko et al.	40 km	5 cm
	Deskew filter [21,24,34]	Du et al.	80 km	80 cm
	Narrow linewidth laser [15]	Geng et al.	95 km	Not mention
	Narrow linewidth laser [16]	Ding et al.	170 km	200 m
Short tuning range method	dynamic OFDR [37]	Arbel et al.	10 km	Not mention
	Fractional Fourier Transform [38]	Shiloh et al.	20 km	2.8 m
	TGD-OFDR [17]	Liu et al.	110 km	1.64 m
	Kerr phase-interrogator [18]	Baker et al.	151 km	11.2 cm

**Table 2:** Performance summary of DVOFS based on OFDR.

Method	Author	Sensing Range	Spatial Resolution	Frequency
CCSA method for the spatial domain signals [53]	Ding et al.	12 km	5 m	2 kHz
CCSA method for the optical frequency domain signals [54]	Liu et al.	40 km	11.6 m	No
M-CCSA method for the optical frequency domain signals [55]	Ding et al.	92 km	13 m	No
TGD-OFDR [56]	Wang et al.	40 km	Not mention	600 Hz
Dynamic OFDR [57]	Arbel et al.	101 km	<10 m	600 Hz
